# Metastable structures and size effects in small group dynamics

**DOI:** 10.3389/fpsyg.2014.00699

**Published:** 2014-07-10

**Authors:** Rosapia Lauro Grotto, Andrea Guazzini, Franco Bagnoli

**Affiliations:** ^1^Psychology and Psychiatry Section, Department of Health Sciences and Center for the Study of Complex Dynamics, University of FlorenceFlorence, Italy; ^2^VirtHuLab, Department of Education and Psychology and Center for the Study of Complex Dynamics, University of FlorenceFlorence, Italy; ^3^Department of Physics and Astronomy and Center for the Study of Complex Dynamics, University of FlorenceFlorence, Italy; ^4^Istituto Nazionale di Fisica Nucleare, Sezione di FirenzeFlorence, Italy

**Keywords:** small group dynamics, therapeutic group, complex systems, Sociophysics, Bion basic assumptions

## Abstract

In his seminal works on group dynamics Bion defined a specific therapeutic setting allowing psychoanalytic observations on group phenomena. In describing the setting he proposed that the group was where his voice arrived. This physical limit was later made operative by assuming that the natural dimension of a therapeutic group is around 12 people. Bion introduced a theory of the group aspects of the mind in which proto-mental individual states spontaneously evolve into shared psychological states that are characterized by a series of features: (1) they emerge as a consequence of the natural tendency of (both conscious and unconscious) emotions to combine into structured group patterns; (2) they have a certain degree of stability in time; (3) they tend to alternate so that the dissolution of one is rapidly followed by the emergence of another; (4) they can be described in qualitative terms according to the nature of the emotional mix that dominates the state, in structural terms by a kind of typical “leadership” pattern, and in “cognitive” terms by a set of implicit expectations that are helpful in explaining the group behavior (i.e., the group behaves “as if” it was assuming that). Here we adopt a formal approach derived from Socio-physics in order to explore some of the structural and dynamic properties of this small group dynamics. We will described data from an analytic DS model simulating small group interactions of agents endowed with a very simplified emotional and cognitive dynamic in order to assess the following main points: (1) are metastable collective states allowed to emerge in the model and if so, under which conditions in the parameter space? (2) can these states be differentiated in structural terms? (3) to what extent are the emergent dynamic features of the systems dependent of the system size? We will finally discuss possible future applications of the quantitative descriptions of the interaction structure in the small group clinical setting.

## 1. Group as a therapeutic device: a brief historical introduction to the structural and dynamic approach

In his 1909 work the sociologist Charles H. Cooley distinguished primary groups, where the individuals perceive themselves as members of a unified collectivity and share a common system of values and practices, and secondary groups, that meet in order to reach a specific target.

In the first two decades of the 20th century different health care professionals in the U.S. building up on Cooley's perspective adopted a collective setting within their therapeutic practices (Bertani et al., 2002).

Joseph Pratt, as a M.D. at the Massachusetts General Hospital in Boston, in 1905 started a weekly group activity with 15 patients suffering from tuberculosis: reading activities and discussions about the illness condition were proposed in order to provide education and psychological support to the participants. Edward Lanzell proposed in 1919 a group talking cure for his psychotic patients. Julius Metzl adopted the same method to treat alcohol dependence. In the same period Trigant Barrow, at that time an outstanding personality of the newborn psychoanalytic movement in the U.S. started to experiment group therapy with neurotic patients. The perspective adopted by Barrow is relevant for its theoretical as well as clinical implications (Burrow, 1927): he was deeply involved in exploring the disrupting effects of the authority position in the psychoanalytic relationship and attempted first to overcome it by experimenting, together with his collaborator, Clarence Shields, reciprocal analysis. They then moved to group psychotherapy in order to obtain a structural rearrangement of the classical asymmetry of the dyadic setting in terms of a more inclusive, egalitarian framework. This was the down of group analysis and the foundation of a new theoretic perspective in which the structure of the human mind is grounded in group interaction and social representations (Galt, 1958, 1991). Further development in the direction of exploiting groups in order to investigate human behavior was achieved by Jacob Levi Moreno with the application of socio-metric techniques and with the shift to active group methods (Moreno, 1951).

Foulkes and Bion, around and after the Second World War in England, produced the first comprehensive systematization of the methodology of group-analysis (Foulkes, 1984) and of the paradigm of the small group with analytic function (Bion, 1961). Here we are mainly interested in Bion's approach, as he explores the structural nature of the “emergent” phenomena that can be observed in the clinical small group setting. With this respect, Bion's theory of small group dynamics can be seen as complementing the Freudian description of large group dynamics: Freud assumed that libidic bonds structure and support crowd phenomena, via the identification of the Leader as the Ideal of the Ego of the members of the group (Freud, 1921).

In describing the psychoanalytic function of the small group clinical setting, Bion stated that in his experience the group was where his voice arrived, a physical limit that is usually assumed to correspond to 8–12 people (Neri, 2011). Bion's approach is considered a psychotherapy “of” the group (in opposition to a psychotherapy performed within the group) as the group behaviors and its unconscious bases are seen as the target of the psychotherapeutic intervention. In fact he introduces a theory of the groupal aspects of the mind in terms of proto-mental states. These are individual mental states that spontaneously evolve into collective psychological states that are the proper observandum in this clinical setting. The collective states at issue correspond to some types of cognitive/emotional experiences that can be detected and described by the analyst. Bion called them basic assumptions, a term that is used in structural anthropology to describe a minimal set of implicit assumptions about the world that renders intelligible the culture of a given group or community under study. They are supposed to be characterized by a series of features: (1) they emerge as a consequence of the natural tendency of (both conscious and unconscious) emotions to combine into structured group patterns; (2) they have a certain degree of stability in time; (3) they tend to alternate so that the dissolution of one is rapidly followed by the emergence of another; (4) they can be described, in qualitative terms, according to the nature of the emotional mix that dominates the state, in structural terms by a kind of typical leadership pattern, and in cognitive terms by a set of implicit assumptions that are helpful in explaining the group behavior. For example, in the fight-flight basic group, the group behaves as if there was an enemy to fight or to flight away from, and as a consequence, appears to be in search of a leader that would be good in identifying such an enemy; in the dependence basic group, the group experiences a set of intense wishes to find an idealized leader that would solve all the group's problems and so on In Bion's view this is not the only relevant way to describe the analytic group's behavior, as the group can also function in a truly cooperative and rational way to fulfill the overt aim of reaching an insight about its own dynamics, with the help of the therapist. But Bion's view is that most of the time the group is dominated by the basic group dynamic, so that an effective, often painful, effort, based on the analytic clarification work, must be sustained in order to produce a real creative development in the state of the group. Although decades of work within this paradigm produced a somewhat more balance view about group dynamics (see for example, Correale, 2006), the fundamental view that the small group dynamics can be described in terms of coherent collective states has never been questioned in formal terms.

Here we propose to adopt a very simple formal model of human interaction and small group dynamics in order to investigate the structural constraints that should support the described phenomenology, in an attempt to address the following issues: (1) are metastable collective states allowed to emerge in the model and if so, under which conditions in the parameter space? (2) can these states be differentiated in structural terms? (3) to what extent are the emergent dynamic features of the systems dependent of the system size? A word of caution is required with respect to the nature of the model we adopt to describe interactions in the group. This is a model derived from a parallel line of investigation in Sociophysics, and therefore, it is not a model derived from psychoanalytic assumptions. Nevertheless, in line with the emergent and structural nature of the phenomena we would like to simulate, and resting on a classical universality assumption, we expect that it can provide a meaningful description of the coherent behaviors in small group dynamics. Our implicit assumption is therefore that in order to study and model groups behaviors in clinical settings it is useful to first consider the basic dynamic behavior of a set of interacting subjects, as approximated by the simulations.

### 1.1. A multidisciplinary approach to the study of the human group dynamics

**Figure 1:**
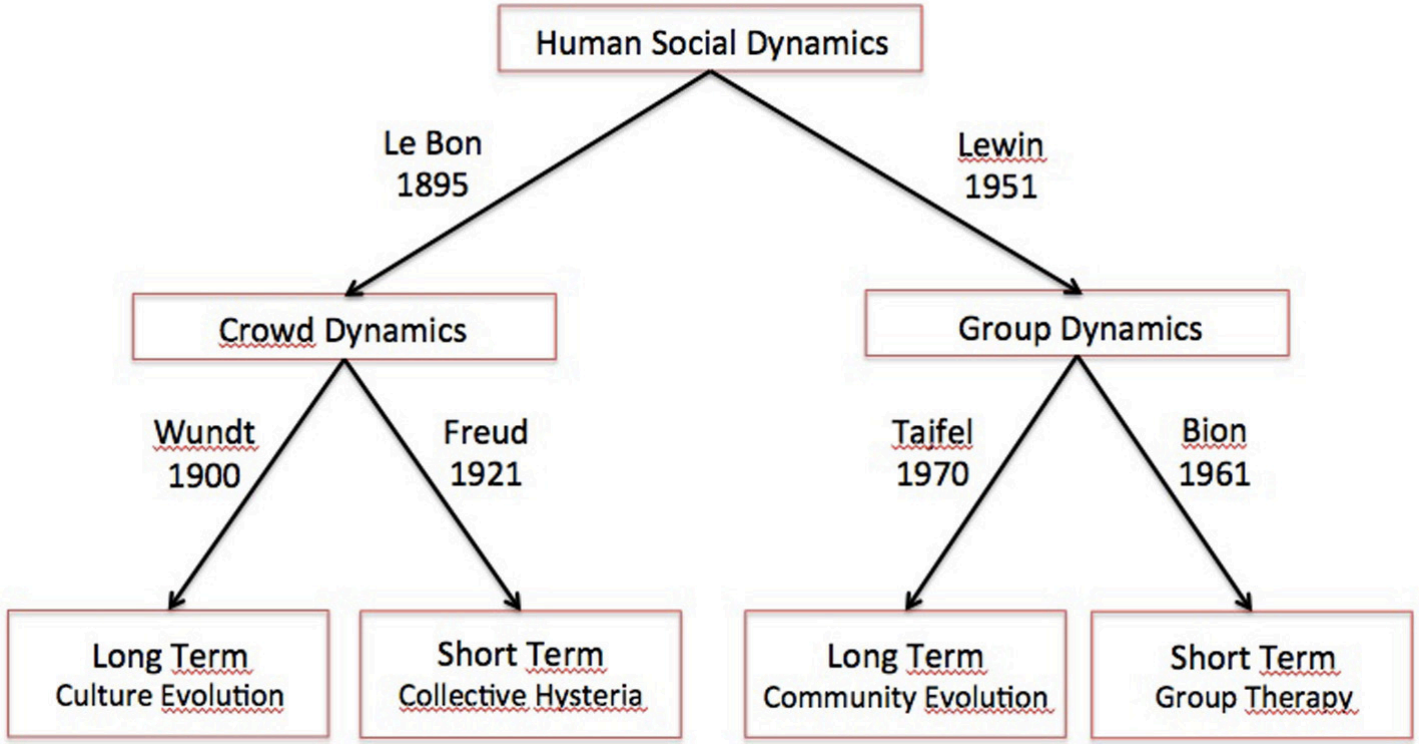
**A classification of the human social dynamics**. A possible and very coarse grained picture describing the classification of human collective dynamics, provided by the classical psychological literature.

A possible and very coarse grained picture describing the classification found in the classical psychological literature of the human collective dynamics is reported in Figure 1.

The recent and fruitful convergence between psychology and complex system science, already provided a new generation of mathematical models and frameworks to study the cognitive group dynamics. In order to reduce the complexity of the system taken into account (i.e., the human groups) the common and fundamental step of such research has been to identify a minimal set of microscopic variables, that capture the relevant mesoscopic representation of the macroscopical dynamics under scrutiny.

Among the large number of disciplines that have been attracted from the study of the human collective dynamics during the last century, Sociophysics represents one of the most powerful paradigm to characterize many relevant collective phenomena, such as culture dissemination, language evolution, spreading of opinions, social norms, credences and beliefs (Lorenz, 2007).

By adopting a multidisciplinary perspective incorporating psychology, sociology, physics of complex systems and computer sciences, Sociophysics developed a modeling approach to reach the mesoscopic approximation of the human systems that is able to capture the interactions between microscopical processes (e.g., psychological and cognitive models and theories), and the macroscopical and observable relevant dimensions (e.g., behaviors, opinions, social norms, and their dynamical features).

In a previous paper (Bagnoli et al., 2008b), we introduced a simple mathematical model describing the opinion dynamics within a group of artificial agents. The agents were equipped with a simple model implementing the cognitive dissonance theory of Festinger (Festinger, 1962), in order to describe, in an effective way, the dynamical interaction between the incoming (i.e., new/external) information and the knowledge of the decision-maker.

## 2. Theory and methods

### 2.1. The agents and the parameters

The fundamental building blocks of our framework are defined agents (or nodes), and links, representing respectively the subjects enrolled in the group dynamics and the quality of their relationships (i.e., affinity). The agents and their links are detailed in our model by means of two fundamental parameters, respectively labeled *opinion* of an agent (*O^t^_i_*), and affinity between two agents (α*^t^_ij_*). The environmental features, i.e., the other free parameters of the model, have been directly inspired from the sociophysical literature and are assumed as standards of the framework. Such parameters are: the convergence parameter μ, representing the average degree of convergence in in opinion/behavior after an effective interaction with another agent, and here set to the standard value of 0.5. The critical opinion (Δ*O_c_*) and affinity (α*c*) thresholds, representing respectively a sort of cultural related *Openness of Mind* and *Average Tolerance* toward the others. And finally the *Social Distances Space* (*D^t^_ij_*), described later in Section 2.3 and the *Social Temperature* (*KT*), incorporating respectively a dimension related with a basic probability of interaction between two subject *i* and *j*, and the degree of mixing (i.e., the probability of meeting a very distant subject on our social distances space) given a certain social setting.

The role and the theoretical meaning of the parameters considered by our model, have been quite well studied and described in the sociophysical literature (Lorenz, 2007). Nevertheless a brief qualitative description of their role is provided in order to clarify the key features of their interplay. The two principal variables used to describe the system dynamics (i.e., order parameters), represents the *opinion* of an agent at a certain time *t* (*O^t^_i_*), and the strength of his relations with the others (i.e., affinity, α*^t^_ij_*). The adoption of a numerical encoding for such dimensions allows us to define a sort of *distance* between subjects in terms of opinion, or expressed behavior, so taking into account the opinion space of the group and the relative position of an actor within it. With respect to the affinity between subjects (α*^t^_ij_*), such a parameter allows to describe in a continuous way (i.e., α*^t^_ij_* ∈ (0,1)) what we could label as the *strength of a relation*, or from another point of view, the influence a subject *i* is subjected to with respect to another subject *j*. As a consequence because of the Equation 1, an affinity close to 0 between two subject would determine a null convergence in the opinion space after an encounter between them (i.e., Δ*O^t−1^_ij_* = Δ*O^t^_ij_*). While an affinity close to 1 would produce a convergence between the agents (i.e., Δ*O^t−1^_ij_* > Δ*O^t^_ij_*), possibly making them *agree* to the same opinion/behavior. In other words with the previous parameters (i.e., *O^t^_i_* and α*^t^_ij_*) we introduce a formal description of the psychological field determined by the group.

The parameters introduced to mimic the dynamics among humans, respectively the convergence parameter μ, and the thresholds of the model Δ*O_c_* and α*c*, have the role to determine the mechanism affecting the inner state of the subjects after an encounter. The convergence parameter μ has the simple role of determining the degree of convergence, namely the maximum percentage of the distance between two interacting subject *i* and *j* that could be traveled by one subject toward the other. As such parameters has been very well studied in sociophysical terms (Weisbuch et al., 2002), proving how his role is affecting only the fastness of the convergence and not the qualitative final state of the system, it is nowadays always set to a convenience value of 0,5. In this way such parameters is maintained in the model just to make it more readable in sociophysical terms, but actually treated as a constant. On the other hand to have a μ with a value of 0.5 means to simulate a situation in which, in the best case, two interacting subjects *i* and *j* characterized by a sufficient degree of affinity (i.e., α*^t^_ij_*, α*^t^_ji_* > α_*c*_), converge after an encounter on the same final opinion, spanning the same distance in the opinion space. The role of the two thresholds of the model (i.e., Δ*O_c_* and α*c*) is fundamental to mimic the effect of the cognitive dissonance on the evolution of the group. The threshold defined on the opinion space Δ*O_c_*, labeled as *Openness of Mind*, determines the maximum degree of distance in opinion tolerated by a subject in order to increase his affinity toward such interactor. A distance Δ*O^t^_ij_* > Δ*O_c_* between two interacting subjects determines a reduction of the affinity between the subjects, while the opposite case would have the opposite effect increasing their affinity. The same coupling is proposed for what concern the affinity threshold α*c*, if the affinity between two interacting subjects is greater than the threshold (i.e., α*^t^_ij_* > α_*c*_) then the two subjects converge in opinion, so reducing their distance Δ*O^t^_ij_*. The key mechanism implementing in the model the cognitive dissonance effect is represented by the coupling between the thresholds and the parameters evolution (2 and 1), i.e., the opinion threshold determines the affinity dynamics 2, and the affinity threshold drives the opinion dynamics 1. The consequence can be summarized as follows: the human tendency is to get along with people sharing our same opinion/behavior, or supporting our same issues, without considering or actively not supporting the others.

The last fundamental ingredient of our numerical recipe is represented by the dynamics of the encounters/interactions between the subjects. This fundamental aspect of a group dynamics is one of the classical weak aspects of the sociophysical approximation. In particular for what concern small group dynamics, taking place for short periods of time, the small number of possible interactions make such events very impacting on the overall dynamics of the group. In order to increase the ecologicity of our model, we represented the subjects belonging to the group as characterized by a *Social Distance* representing the probability to observe an interaction between them. To build such distance we started from two simple considerations, the first is that humans not have only random interactions, but at the contrary are used to affect a lot the probability of their encounters, choosing where to go, what to say and to who express their opinion. In order to introduce such an aspect, we implicitly stated in our Equation 3 that a subject would like to interact more likely with a *friend* close to him in the opinion/behavior space (i.e., Δ*O^t^_ij_* → 0), and linked with him by an high affinity (i.e., α*^t^_ij_* → 1).

In order to introduce the stocastic or random interactions always taking place during a group dynamics, in the Equation 4 the probability of an encounter is *thermalized* or *perturbed* by a gaussian noise with mean 0 (i.e., the sum of positive and negative noise/displacements on the social distances space of a subject is equal to 0). The gaussian perturbation determines that after a completely random extraction of a subject *i* (i.e., namely the first interactor), every subject *j* within his social space is moved in the two possible directions (i.e., far or close) of a term equal to the noise. A different noise term is extracted from the same distribution and added to the distance between *i* and *j*, and at the end the closest agent to *i* is selected for the interaction. Using such a mechanism we can simulate different scenarios characterized by different degrees of *social temperature* or social mixing, just by tuning the standard deviation of the gaussian noise distribution (i.e., σ_*Noise*_ = *KT*). As a consequence we have that there is always a probability different from 0 to observe any possible interaction, and that we can tune the degree of mixing in order to obtain the same probability for every possible interaction (i.e., high social temperature), or to give a greater relevance to the initial distances space (i.e., low social temperature).

Finally, by means of our framework, we are able to describe a subject enrolled in a group experience as a trajectory on a multidimensional space, describing at the same time his microscopical features (i.e., his opinion and his community), as well as the macroscopical factors affecting his dynamics.

Our numerical simulation are devoted to investigate the effect of the size of the group within our theoretical approximation, in search of any macroscopical feature, related with the free parameters of the model (i.e., *affinity* and *opinion thresholds*, *social temperature*, and *group size*) that could suggest that a phase shift is present in the collective dynamics.

At the beginning of each simulation the initial conditions of the system are set simply by assigning a random uniform distributed opinion (*O^0^_i_*), ranging in (0,1), as well as a random affinity (α*^0^_ij_*) value for each dyads, with *alpha^0^_ij_* ≠ α^0^_*ji*_. In this way the vector *O^t^_i_* and the matrix *A^t^_ij_* are defined as respectively the opinion and the affinity spaces of the system.

Each iteration represents an encounter where two agents are extracted with a certain rule and interact, updating their parameters (i.e., *opinion* and *affinity*) accordingly with the rules of the model.

The macroscopical dynamics of the system can be considered in a stable/equilibrium (i.e., or metastable) state, if a relevant order parameter of the system (e.g., a macroscopical variable of interest, such as the number of sub-communities acting within the group) reaches a temporal stability, i.e., does not change for a long time and/or for a large number of subsequent interactions/communications. In our study we consider the final number of sub-communities (i.e., clusters) that emerge in the simulation along the time.

Finally, in order to get an insight about a possible critical size of the human groups, distinguishing between *crowd* and *small group* dynamics, we adopted as control parameter of our simulation the size of the group (*N*), and studied its effects on the dynamical behaviors of the vector *O_i_* and of the matrix *A_ij_*.

### 2.2. The model

The mathematical model we studied in Bagnoli et al. (2008a,b); Carletti et al. (2009) incorporated the Cognitive Dissonance theory of Leon Festinger in order to detail the mechanics of the evolution of the agents' parameters after the encounters. Briefly, when two agents meet, their opinions converge if between the agents the affinity level is larger than the *critical affinity threshold* (i.e., α*^t^_ij_* > α_*c*_), remaining still otherwise (1). At the same time the coupled equation evolving the affinity between subjects (2), determines an increasing of α*^t^_ij_* if the absolute value of the difference in opinion between the two subjects (Δ*O^t^_ij_*) is smaller than the *Critical Opinion Threshold* Δ*O_c_*, otherwise the affinity α*^t^_ij_* is reduced, always ranging between 0 and 1.

(1) $$O_{i}^{t+1}=O_{i}^{t}+\mu \Delta O_{ij}^{t}\frac{\tanh\left(\beta (\alpha_{ij}^{t}-\alpha_{c}^{t})\right)}{2}$$

(2) $$\alpha_{ij}^{t}+1=\alpha_{ij}^{t}+(1-\alpha_{ij}^{t})\alpha_{ij}^{t}\tanh\left(\beta (\Delta O_{c}-\Delta O_{ij}^{t})\right)$$

where *O^t^_i_* is the Opinion (i.e., or a behavior) shown by a subject *i* at the time *t*, with *O* ∈ (0,1). While α*^t^_ij_* represents the strength of the relation between the subjects *i* and *j*, at the time *t*, with α ∈ (0,1). More in detail Δ*O^t^_ij_* represents the difference (or distance) in Opinion/Behavior or Psychological State between two subjects *i* and *j* in a certain moment *t*. Of course this parameter allows to introduce a threshold, Δ*O_c_*, to represent a sort of “*Openness of Mind*” of the group, or in other words, the average availability of the subjects to change their feelings toward those interactors characterized by a very different Psychological State/Opinion/Behavior with respect to them. The parameters μ and β, set respectively to values 0.5 and 1000, are just devoted to determine the speed of the convergence of the simulations, and do not alter the final qualitative results (Weisbuch et al., 2002).

The model implements different psychological assumptions, ranging from the Cognitive Dissonance of Festinger (1962), to the Psychological Field of Lewin (1951), and the Social Impact Theory of Asch and Sherif (Asch, 1956; Sherif and Hovland, 1961).

Within our model the dynamics of the evolution of the psychological state is coupled with the evolution of the affinity between the subjects belonging to the same group dynamics. More in details, the two hyperbolic tangent equipping the Equations 1 and 2, introduce two step functions to mimic the cognitive dissonance theory effect. The Psychological State of a subject (*O^t^_i_*) evolves as a consequence of the interaction with another subject, but the magnitude of the effect (i.e., of the change in the State variable) is modulated by the affinity toward that subject α *^t^_ij_* with respect to the critical affinity value (i.e., α_*c*_). In other words, if the subject *i* has a strong “affective” link with the subject *j*, he would change his Psychological State easily. At the same time the evolution of the affinity between subjects is controlled by the critical difference in psychological state, or Δ*O^t^_ij_* parameter. The Equation 4 couples the evolution of the affinity between *i* and *j*, with their difference in term of Psychological State (i.e., Δ*O^t^_ij_*). In details, if the difference between the Psychological State of two interacting subjects is smaller than a certain critical value, here labeled as Δ*O_c_*, their affinity after the encounter will increase. Obviously the opposite happens if the difference Δ*O^t^_ij_* is greater than Δ*O_c_*.

### 2.3. A numerical recipe for the group simulation

In order to get an effective, and *ecological*, representation of a real human group dynamics, we spent an effort even in the design of the dynamics of interactions among the agents. In our representation of a real dynamics, every subject *i*, at each time step, is equipped with a memory of his past interactions called Social Distances Space of *i*. Within such a dimension should be represented the probability to observe an interaction between the subject *i* and any other subjects belonging to the interaction. A first mathematical approximation of this dimension can be the following:

(3) $$d_{ij}^{t}=\Delta O_{ij}^{t}(1-\alpha_{ij}^{t})$$

the two simple assumptions seeding the equation are the following: a subject has higher chances to iteract both with those who are nearer to him in terms of Psychological State, and with those toward whom he feels a higher affinity.

Once the Social Distances Space is defined, an important and still missing ingredient is the dynamics of the interactions. In order to refine the model we manipulated this phase by introducing a *thermalization phase* representing a certain level of unpredictability of the system; such an ingredient can make every event as a singularity.

**Figure 2:**
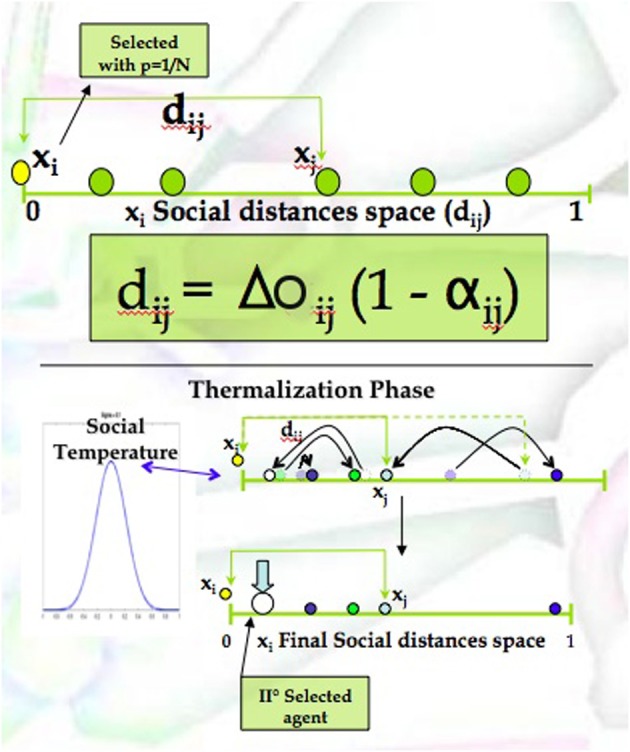
**The social distances space**. Graphical representation of the mathematical recipe used to simulate the encounters dynamics among agents.

The thermalization phase has been structured as follows and illustrated in Figure 2.

- Agent *i* Selection/Extraction:
  In the first step of the recipe an agent *i* is randomly selected from the community (i.e., using a uniform probability distribution).
- *i*-Social Space Thermalization:
  Once the individual *i* is selected, its social distances *d^t^_ij_* with respect to all other individuals are computed and randomly varied with a white random noise η*^t^_i_*, as reported in Equation (4). The standard deviation of the noise (η) is assumed as a control parameter of the system, and because of its power of *mixing* and *shuffling* the system it is labeled as Social Temperature. The resulting social distances space for the subject *i* is, as a consequence, given by the equation:
  (4) $$d_{ij}^{t}=\Delta O_{ij}^{t}(1-\alpha_{ij}^{t})+\eta_{i}^{t}$$
- Agent *j* Selection:
  After the thermalization phase, the nearest agent to *i* is selected as interactor (i.e., *j*).

## 3. Results

In order to study the effect of the size of the group on the spontaneously emerging dynamics, we varied the *N* parameter for different numerical simulation.

**Figure 3:**
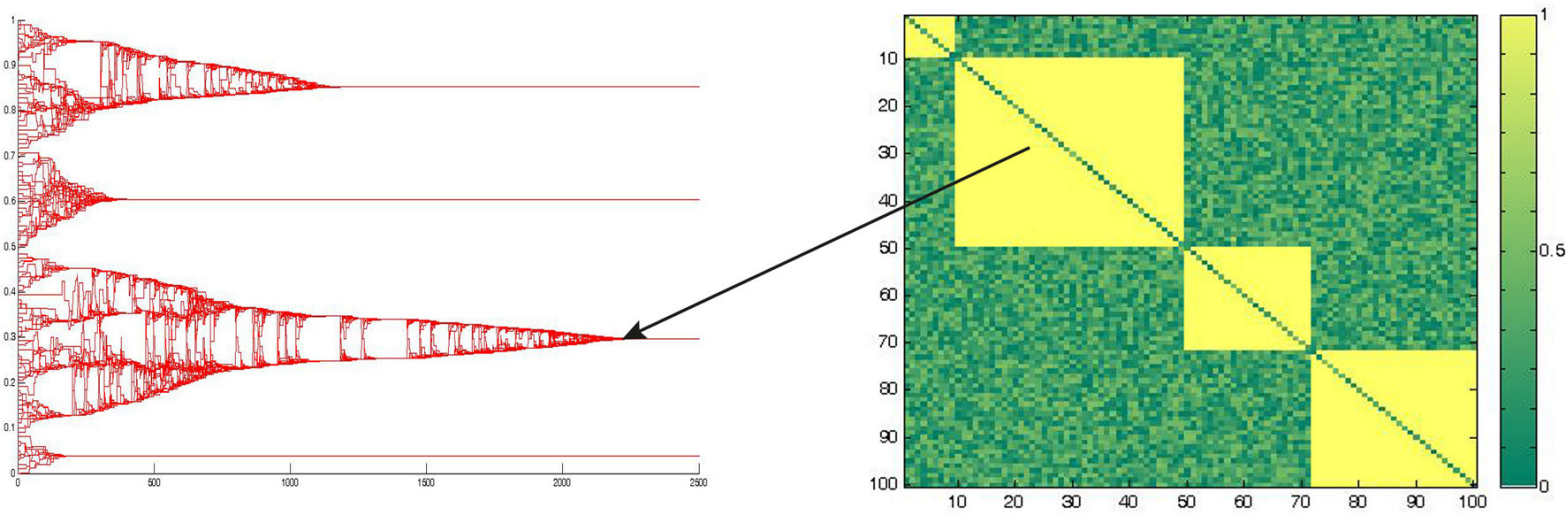
**Stable state of the group dynamics**. An example of stable state condition of the system at the end of a typical simulation run is reported. On the left the opinion space of the group, on the y-axis, is represented with respect to the time on the x-axis. On the right the final affinity matrix is represented, on the axis are reported the nodes and the higher is the affinity between two subjects, the more the correspondent cell/value is yellow.

The model's dynamics is characterized by dynamical equilibrium states, as shown in Figure 3, defined as those state in which the affinity matrix as well as the opinion space do not show any further change in time.

The Figure 3 represents an example of stable state condition, reached by one simulation run. In particular on the left is reported the temporal evolution of the opinion vector *O^t^_i_*, while on the right is shown the final state of the affinity matrix *A^t^_ij_* whit the affinity values ranging from yellow (i.e., large values) to green (i.e., small values). In the particular example reported, four clusters characterize the stable state, and the arrow suggests the correspondence between the two projection of the same system, so that the four clusters can be represented or using the affinity matrix, or considering the opinion space The equilibrium state can be described in terms of final number of clusters, and in terms of amount of time required to reach the stable state in the affinity space, as well as in the psychological state space (i.e., opinion space). Such times of convergence are going to be taken into account, later in this paper, in order to discriminate the two regimes emerging from our results.

**Figure 4:**
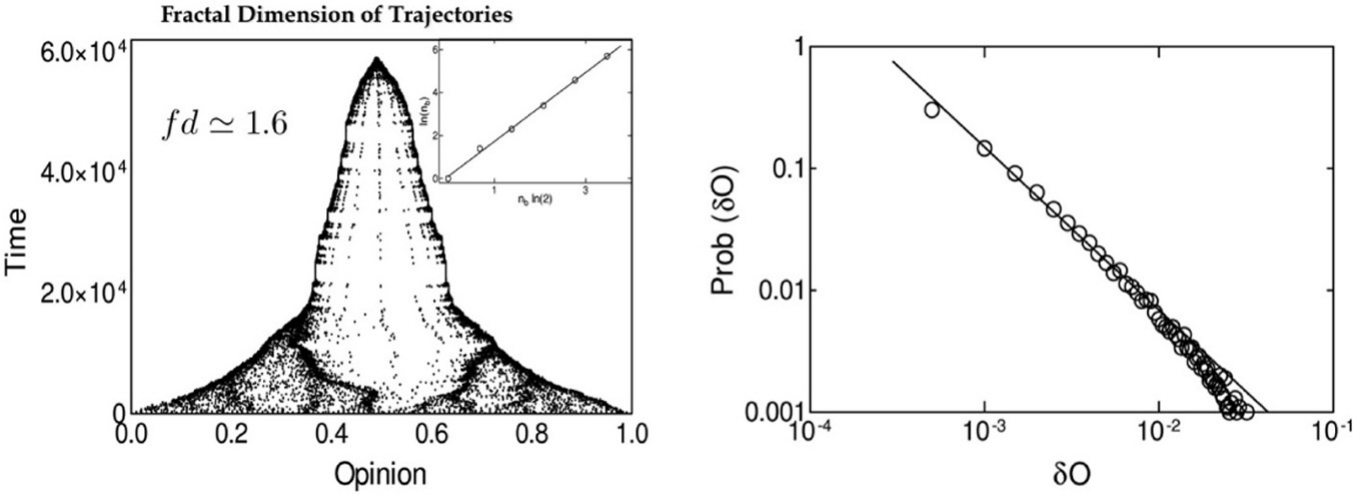
**Complex dynamics features**. Among the interesting features suggesting the existence of a self organizing complex dynamics beyond the systems studied by our model, the figures above report probably the most important. On the left the agents' trajectories for a single simulation are reported and analyzed by mean of the box method, reporting a fractal dimension of such trajectories. On the right the distribution along three decades of the opinion jumps of the agents (i.e., the movements along the opinion space realized by the agents during the entire simulation) is reported using a logarithmic rescaling. The linearity of the interpolating function suggest a power law function controlling such a process.

As it is reported in Figure 4, the numerical simulations of our system suggest the existence of a self critical process. The fractal dimension for our system has been estimated to be *fd* ≃ 1.6, and the probability distribution of the “Psychological State Change” appears to be fitted by a power law distribution.

**Figure 5:**
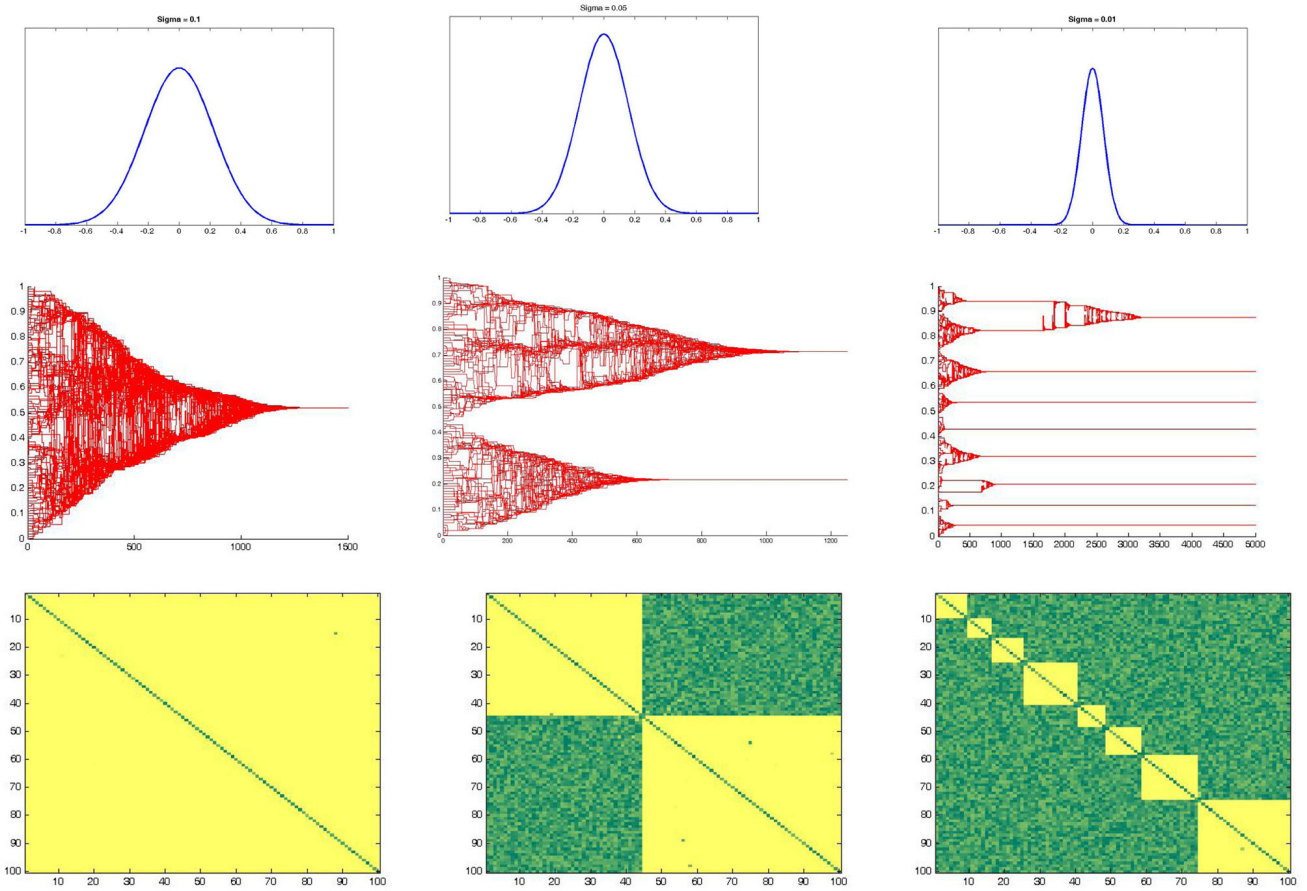
**Social temperature effect**. The three pictures above report the effect of social temperature, which has been defined as the standard deviation of a white gaussian noise with mean equal to 0. Increasing the social temperature (i.e., from right to left) make the final number of clusters decreasing.

The Social Temperature effect is reported in Figure 5, and confirm the classical Sociophysical literature reporting as, the higher is the mixing of the agents' encounter (i.e., an high probability to have an interaction between subjects regardless their initial state or affinity), the greater is the probability to have a single final cluster as equilibrium of the system (i.e., a condition where all the subjects show the same Psychological State and have an high affinity which each other). As a consequence, decreasing the Social Temperature makes more probable to have a fragmented state (i.e., more than one final cluster) as an equilibrium state.

**Figure 6:**
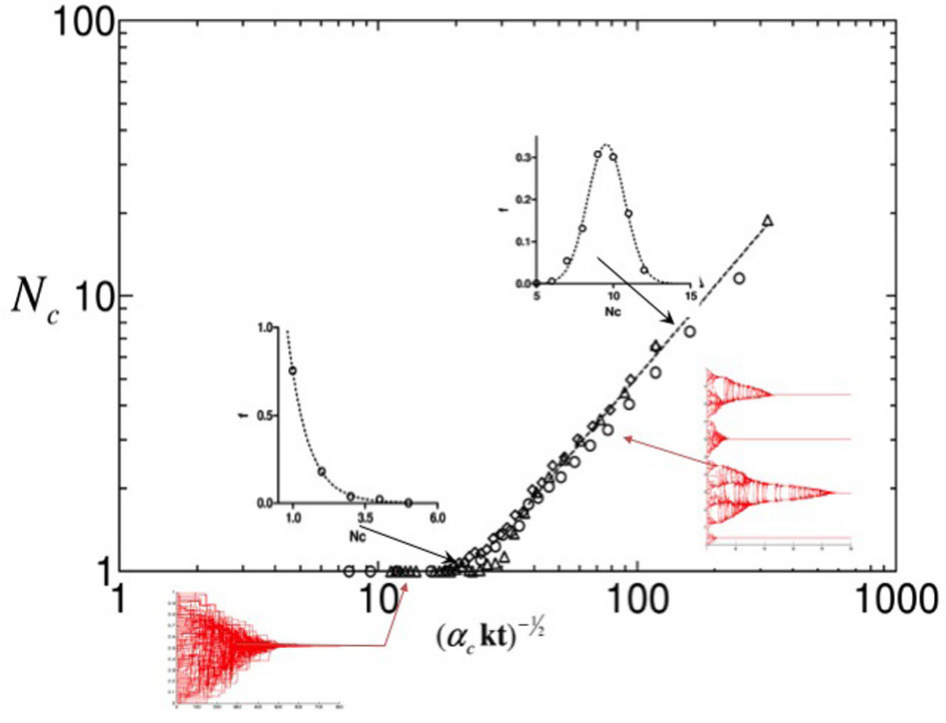
**System phase transition**. The figure reports the average number of clusters as function of the rescaled quantity ${\left(\sigma \alpha_{c}\right)}^{-\frac{1}{2}}$. A phase transition is found at ${\left(\sigma \alpha_{c}\right)}^{-\frac{1}{2}}$ ≃ 20. Above the transition, histograms of the number of clusters are computed and enclosed as insets in the main frame: symbols refer to the numerics, solid lines are fitted interpolation. Here, Δ*Oc* = 0.5.

An appropriate scaling of the numerical simulations' data has shown, in a previous work (Bagnoli et al., 2008b), a second order phase transition on the order parameter related to the number of final cluster (Figure 6). The resulting law describes the relation between the final psychological coherence of a community, the critical affinity shared by the subjects, and the social temperature (*ST*) or degree of mixing (Equation 5). The Equation 5 suggests that the average final number of clusters decreases when the social temperature and the average critical affinities increase (Figure 6).

(5) $$N_{c}=\frac{1}{\sqrt{\alpha_{c}ST}}$$

**Figure 7:**
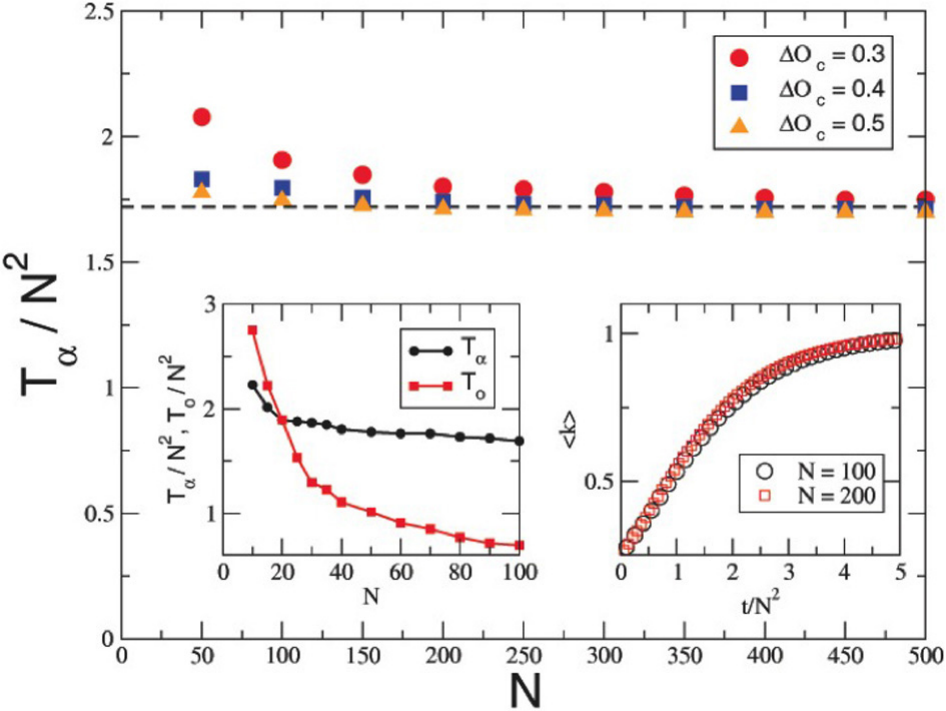
**Size effect on system dynamics**. The main panel of the figure reports how T/N2 vs. N for dierent values of the parameter Δ*O_c_*. The data approach a constant value ($\frac{T}{N^{2}}≃1.72$) clearly indicating that the time of convergence of the anity matrix scales quadratically with the number of agents, in agreement with the theory. The asymptotic value estimated by our theory is 2.19, the discrepancy being therefore quantied in about 15%. Left inset: $\frac{T}{N^{2}}$ and $\frac{Tc}{N^{2}}$ vs. *N* for Δ*Oc* = 0.5. As predicted by the theory and the numerics a crossover is found for groups for which opinions converge slower than the anities: this is the signature of a distinctive dierence in the behavior of small and large groups, numerically we found that this dierence is eective for *N* ≃ 20. Right inset: σ(*k*) vs. $\frac{t}{N^{2}}$ is plotted for two dierent values of *N*. As expected the two curves nicely collapse together.

In order to assess the effect of the group size on the evolution dynamics of the system, we rescaled the convergence times of the two dimensions under scrutiny (i.e., Opinion space and affinity space), with the factor *N*^2^. Such a transformation is sufficient, as shown in the subfigure on the right of the Figure 7, to make the different functions collapsing on the same plane. The upper diagram demonstrates how good is the approximation obtained by the scaling with *N*^2^ of the convergence time of the affinity matrix, increasing the size of the system (*N*), for different critical values of openness of mind. The function suggests a typical value for “large” systems, and a divergence for “small” systems (i.e., groups). Finally, Figure 7 reports, on the left bottom corner subfigure, the two functions representing, respectively, the affinity convergence time (in black), and the opinion convergence time (in red), with respect to the size *N* of the community. In our simulations the two functions cross each other for a value of *N* between 10 and 20. In other words, before such a critical size of the group the affinity matrix (i.e., the representation of the strength of the relationships between subjects) reaches the final state first, training subsequently the Psychological States dynamics. On the contrary, when the size of the system increases, the affinity dynamics become slower than the Psychological States dynamics, and it is this last one that drives the convergence of the affinity matrix once it reaches its stable state.

## 4. Discussion

In our simulation study, we have explored the asymptotic behavior of the group dynamics in a model of interacting agents endowed with an Opinion state and a matrix of Affinity levels that evolve according to a coupled non-linear updating law. The aim of the simulations was to assess the plausibility, in dynamic terms, of the model proposed by Bion in the context of his analytic studies in the small group clinical setting (Bion, 1961). In particular, we wanted to verify the plausibility of the proposed hypothesis that the group dynamics is characterized by the spontaneous and rapid self-organization of coherent states that exhibit a degree of stability in time and that can be described in structural terms by specific patterns in the Opinion and Affinity spaces. These latter variables intend to represent, in the model, the cognitive and affective evolution of the participants taking place via interactions in the group.

The model is characterized by the presence of different control parameters; the dependence of the asymptotic behavior of the dynamics from these parameters and the eventual stability has been analytically explored in the simulations. We would like to stress that this model is not endowed with any *ad hoc* computational mechanism to enhance stability, such as symmetric interactions between the agents. The collective phenomenology is therefore a direct consequence of the interaction dynamics.

The first result that we obtained in the simulations is the emergence of collective coherent states that are quite rapidly stabilized in time. From the plot of the asymptotic Affinity matrices and Opinion states, it is evident that these asymptotic states are characterized by complex patterns of dynamic clustering (See Figure 3); this pattern tends to be simplified only in the presence of the higher levels of Gaussian Noise in the model (See Figure 5). The structure exhibited by the asymptotic state in the Opinion space can be further characterized in terms of a self critical phenomenon with fractal dimension 1.6, as shown in Figure 4.

The second result that we would like to stress, is obtained by considering the speed of convergence of the Opinion and Affinity variables toward their asymptotic values as a function of the group dimension *N* (see Figure 7). We remind that the Opinion and Affinity dynamics are coupled in the model and that the simulations are triggered by assuming random values of the Opinion and Affinity variables at time zero. Nevertheless, the model exhibits a very interesting behavior: when *N* is in the range of less than around 20 units, the convergence of the Affinity matrices is faster than the convergence of the Opinion variables, while the opposite is true for *N* larger than 20. This phenomenology in the simulations is suggestive of the existence of two different dynamic regimen in the model, the first corresponding to the classical small group dynamics and the other corresponding to the classical large group or crowd dynamics. In the small group case, the affective structure of the interpersonal links in the group remains the main determinant of the collective state of the system, while in the case of the large group, or crowd dynamics, the cognitive dimension of the Opinion dynamics is dominating the collective behavior. This is reminiscent of the Freudian hypothesis that an Common Ideal or a shared Value can very easily take the place of the “beloved” Leader in the mass condition (Freud, 1921). Overall the results support well the validity of the distinction between the small group and large group dynamics that is so well established in clinical practice. Furthermore the picture proposed by Bion, that the small group exhibits the tendency to be dominated by collective coherent states emerging from the immediate and incompressible tendency (i.e., named Valence in Bionian terms) of individual cognitive/affective states to coalesce into collective asymptotic metastable patterns, seems to be plausible when considered within a formal non-linear group dynamic approach.

A point that is worthwhile mentioning is that, from the simulations, we see that the group dynamics exhibits a certain degree of stability even in the small group case. As a consequence, the tools on non-linear analysis, together with structural network analysis, can be applied to describe the group's behavior in principle even in ecological settings. The relevant issue is therefore to be able to operationally describe the interacting behavior of the participants in a convenient way. As a first step in this direction, our group is developing a dedicated Virtual Ambient for the study of group interactions (www.complexworld.net/virthulab) in which many relevant aspect of the subjects' interactions can be tracked *in vivo*. We are particularly interested in analyzing small group dynamics under different task constraints (Grotto, 2012a; Guazzini et al., 2012b; Cini and Guazzini, 2013). In the present paper, for example, the simulated condition corresponds to an “ecological” (*ICT-mediated*) situation where the participants can freely interact for a given amount of time (i.e., ICT is used for Information and Communication Technologies). The availability of dynamic and network analyses (that could even be related to an analysis of the content of the exchanged messages in the chat) provides a potentially new way to assess issue such as what is it that makes the leader a leader in the group or under which conditions does the group behave as a whole and why in some other conditions fragmented subgroups do emerge in the self-organization process. A further advantage of these new research perspective is that it provides a very natural way to contrast the classical description of a subject in terms of psychological observables with his or her behavior as a participant in the “ecological” group setting.

### Conflict of interest statement

The authors declare that the research was conducted in the absence of any commercial or financial relationships that could be construed as a potential conflict of interest.

## References

[B1] Asch S. E. (1956). Studies of independence and conformity: I. a minority of one against a unanimous majority. Psychol. Monogr. Gen. Appl. 9, 1–70 10.1037/h0093718

[B2] Bagnoli F. Carletti T. Fanelli D. Guarino A. Guazzini A. (2008a). Birth and death in a continuous opinion dynamics model. the consensus case. Eur. Phys. J. B 64, 285–292 10.1140/epjb/e2008-00297-3

[B3] Bagnoli F. Carletti T. Fanelli D. Guarino A. Guazzini A. (2008b). Dynamical affinity in opinion dynamics modeling. Phys. Rev. E 76, 66105 10.1103/PhysRevE.76.066105 18233896

[B4] Bertani B. Manetti M. Venini L. (2002). Psicologia dei Gruppi, Teoria, Contesti e Metodologie Dintervento. Milano: Franco Angeli

[B5] Bion W. R. (1961). Experiences in Groups. New York, NY: Basic Books 10.4324/9780203359075

[B6] Burrow T. (1927). The Social Basis of Consciousness. New York, NY: Harcourt, Brace

[B7] Carletti T. Fanelli D. Guarino A. Guazzini A. (2009). Meet, discuss and trust each other: large versus small groups, in Artificial Life and Evolutionary Computation: Proceedings of Wivace 2008, eds Serra R. Villani M. Poli I. (Singapore: World Scientific), 213–224

[B8] Cini A. Guazzini A. (2013). Human virtual communities: affinity and communication dynamics. Adv. Comp. Sci. 16, 1350034–1–1350034–24 10.1142/S0219525913500343

[B9] Correale A. (2006). Area Traumatica e Campo Istituzionale. Roma, QLD: Borla

[B10] Festinger L. (1962). A Theory of Cognitive Dissonance. Redwood City, CA: Stanford university press

[B11] Foulkes S. H. (1984). Therapeutic Group Analysis. London: Karnac Books

[B12] Freud S. (1921). Group psychology and analysis of the ego. S.E. London Hogarth 18, 65–143

[B13] Galt A. (1991). The phenomenology of normality in the context of Trigant Burrows's group analysis. J. Human. Psychol. 31, 95–113 10.1177/0022167891311009

[B14] Galt W. E. (1958). A Search for Man's Sanity: The Selected Letters of Trigant Burrow. New York, NY: Oxford University Press

[B15] Guazzini A. Bagnoli F. Carletti T. Vilone D. Lauro Grotto R. (2012a). Cognitive network structure: an experimental study. Adv. Comp. Sci. 15, 12584–12599 10.1142/S0219525912500841

[B16] Guazzini A. Cini A. Lauro Grotto R. Bagnoli F. (2012b). Virtual small group dynamics: a quantitative experimental framework. Rev. Psychol. Front. 1, 10–17 Available online at: http://www.academicpub.org/fpbs/paperInfo.aspx?PaperID=1359

[B17] Lewin K. (1951). Field Theory in Social Science. New York, NY: Harper and Row

[B18] Lorenz J. (2007). Continuous opinion dynamics under bounded confidence: a survey. Int. J. Mod. Phys. C 18, 1819–1838 10.1142/S012918310701178923028458

[B19] Moreno J. L. (1951). Sociometry, Experimental Method and the Science of Society. An Approach to a New Political Orientation. New York, NY: Beacon House

[B20] Neri C. (2011). Gruppo. Roma, QLD: Borla

[B21] Sherif M. Hovland C. I. (1961). Social Judgment: Assimilation and Contrast Effects in Communication and Attitude Change. New Haven, CT: Yale University Press

[B22] Weisbuch G. Deffuant G. Amblard F. Nadal J. (2002). Meet, discuss, and segregate. Complexity 3, 55–63 10.1002/cplx.1003112546989

